# Structural and Thermodynamic Understandings in Mn‐Based Sodium Layered Oxides during Anionic Redox

**DOI:** 10.1002/advs.202001263

**Published:** 2020-07-02

**Authors:** Seok Mun Kang, Duho Kim, Kug‐Seung Lee, Min‐Seob Kim, Aihua Jin, Jae‐Hyuk Park, Chi‐Yeong Ahn, Tae‐Yeol Jeon, Young Hwa Jung, Seung‐Ho Yu, Junyoung Mun, Yung‐Eun Sung

**Affiliations:** ^1^ Center for Nanoparticle Research Institute for Basic Science (IBS) Seoul 08826 Republic of Korea; ^2^ School of Chemical and Biological Engineering Seoul National University (SNU) Seoul 08826 Republic of Korea; ^3^ Department of Mechanical Engineering Kyung Hee University Yongin 17104 Republic of Korea; ^4^ Beamline Department Pohang Accelerator Laboratory (PAL) Pohang 37673 Republic of Korea; ^5^ Department of Chemical and Biological Engineering Korea University 145 Anam‐ro Seongbuk‐gu Seoul 02841 Republic of Korea; ^6^ Department of Energy and Chemical Engineering Incheon National University (INU) Incheon 22012 Republic of Korea

**Keywords:** anionic redox, cathodes, sodium ion batteries, two‐phase reactions

## Abstract

A breakthrough utilizing an anionic redox reaction (O^2−^/O^n−^) for charge compensation has led to the development of high‐energy cathode materials in sodium‐ion batteries. However, its reaction results in a large voltage hysteresis due to the structural degradation arising from an oxygen loss. Herein, an interesting P2‐type Mn‐based compound exhibits a distinct two‐phase behavior preserving a high‐potential anionic redox (≈4.2 V vs Na^+^/Na) even during the subsequent cycling. Through a systematic series of experimental characterizations and theoretical calculations, the anionic redox reaction originating from O 2p‐electron and the reversible unmixing of Na‐rich and Na‐poor phases are confirmed in detail. In light of the combined study, a critical role of the anion‐redox‐induced two‐phase reaction in the positive‐negative point of view is demonstrated, suggesting a rational design principle considering the phase separation and lattice mismatch. Furthermore, these results provide an exciting approach for utilizing the high‐voltage feature in Mn‐based layered cathode materials that are charge‐compensated by an anionic redox reaction.

## Graphical abstract

P2‐type Na_0.60_Li_0.20_Mn_0.80_O_2_ undergoes a reversible two‐phase reaction at high voltage (≈4.2V vs Na^+^/Na), in which oxygen ion contributes the charge compensation. The detailed reaction mechanism is thoroughly investigated through experimental and theoretical analyses including in situ XRD and XAS observations. The combined study provides a rational material design for the reversible two‐phase reaction.

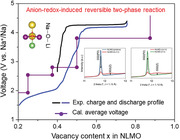

## 1 Introduction

In conventional transition metal (TM) layered oxide cathode materials for lithium‐ion batteries (LIBs) and sodium‐ion batteries (SIBs), charge compensation during the intercalation/deintercalation process relies on changes in the oxidation states of the transition metal ions.^[^ ^1^, ^2^, ^3^ ^]^ For example, in LiCoO_2_, which is a typical layered oxide cathode material in LIBs, the oxidation state of cobalt varies between 3+ and about 3.5+ during the charge/discharge cycles.^[^ ^4^ ^]^ Therefore, enormous research efforts have focused mainly on modifying TM ions to increase the energy density of cathode materials. However, this strategy is limited by the amount of TM ions in the compounds.

In recent years, it has been reported that oxygen ions can reversibly change their oxidation states, as opposed to the conventional concept of stable oxide ions with a constant oxidation number of −2.^[^ ^5^ ^]^ Thanks to these findings, a new paradigm of anionic redox reactions for charge compensation has led to improved capacity of Li‐rich layered oxide compounds via the introduction of another redox center for cathode materials.^[^ ^6^, ^7^ ^]^ Among them, Li‐rich layered manganese‐based oxides, *x*Li_2_MnO_3_∙(1‐*x*)LiTMO_2_, are mainstream classes, and they are composed of two parts: a typical layered oxide (LiTMO_2_) and a Li‐rich layered oxide (Li_2_MnO_3_). The structure of Li_2_MnO_3_, which is also denoted as Li[Li_1/3_Mn_2/3_]O_2_, is similar to that of typical LiTMO_2_, but 1/3 of the Mn ions are replaced by Li ions, resulting in a structural distortion from a hexagonal system to a monoclinic system induced by the in‐plane honeycomb‐type ordering where the Li ion is surrounded by six Mn ions.^[^ ^8^ ^]^ In earlier studies of Li‐rich layered oxides, Li_2_MnO_3_ was recognized as electrochemically inactive due to the stable electron configuration of the initial Mn^4+^ (*t*_2g_^3^ *e*_g_^0^), which is not feasible to further oxidation in an octahedral geometry. However, Li_2_MnO_3_ exhibits a considerable charge capacity with a long plateau upon extraction of Li^+^ at the high voltage at 4.5 V (vs Li^+^/Li), involving the oxidation reaction of the lattice oxygen ions in Li_2_MnO_3_.^[^ ^9^ ^]^ The mechanism of the oxygen redox reaction in Li‐rich layered oxide compounds is related to the unhybridized O 2p orbitals in the Li_Li_‐O‐Li_TM_ configuration (where a Li_Li_ ion is located in the Li layer and the other (Li_TM_) is located in the TM layer) because of the relatively small overlap with the Li 2s orbital.^[^ ^10^, ^11^ ^]^

In addition, the oxygen redox reaction in SIB layered oxide cathode materials has been intensively studied in recent years. An earlier study^[^ ^12^ ^]^ suggested a possible contribution of lattice oxygen ions to a charge compensation reaction to account for unusual excess capacity of P2‐type Na_0.78_Ni_0.23_Mn_0.69_O_2_ during the first charge process. More recently, a possible anionic redox activity in Na[Li_1/3_Mn_2/3_]O_2_ by first‐principle calculations was predicted^[^ ^13^ ^]^; as a result, the initial charge capacity (≈150 mAh g^−1^) with a large plateau at 4.2 V (vs Na^+^/Na) of the synthesized Na‐deficient Na_0.76_Li_0.25_Mn_0.75_O_2_ phase relies solely on the oxygen redox activity. And, it was demonstrated that a lone pair of O 2p‐electrons (unhybridized O 2p‐electron) in the linear Na—O—Li configuration participates in the charge compensation during charging. Furthermore, Yabuuchi et al.^[^ ^14^ ^]^ reported that a P2‐type Na_2/3_[Mg_0.28_Mn_0.72_]O_2_ without alkali–metal ions in a transition metal layer exhibited a large reversible capacity beyond the theoretical capacity via only Mn^3+/4+^ redox reactions. Maitra et al.^[^ ^15^ ^]^ found that the excess capacity originated from the oxygen redox reaction of P2‐type Na_2/3_[Mg_0.28_Mn_0.72_]O_2_. This research interest in the anionic redox reaction at the high voltage has triggered discovery of various high‐energy‐density SIB cathode materials to date.^[^ ^16^, ^17^, ^18^, ^19^, ^20^ ^]^

In the abovementioned studies, the anionic (oxygen) redox active layered oxide compounds exhibit a characteristic long charge plateau at the high voltage (4.2 V) during the first charge process, leading to high‐energy‐density cathode materials for SIBs. Subsequently, they show a large voltage hysteresis with loss of the plateau in the following discharge process, resulting in poor energy efficiency.^[^ ^10^, ^14^, ^15^, ^16^ ^]^ On the other hand, among these oxygen redox active materials, Na_0.6_[Li_0.2_Mn_0.8_]O_2_ with a P3‐type layered structure shows an unusual flat discharge plateau at 4.1 V (vs Na^+^/Na), as reported by Du et al.^[^ ^21^ ^]^ This result is clearly distinguishable from the solid‐solution behavior of P2‐type Na*_x_*[Li*_y_*Mn*_z_*]O_2_ compounds^[^ ^22^ ^]^ despite the similar [Li*_y_*Mn*_z_*]O_2_ slab structure. Rong et al.^[^ ^23^ ^]^ also investigated a charge compensation mechanism in P3‐Na_0.6_[Li_0.2_Mn_0.8_]O_2_ that relies solely on an anionic redox reaction exhibiting the reversible charge–discharge plateau in the voltage range of 3.5–4.5 V (vs Na^+^/Na) using a spectroscopic study, and identified the shortening of the O—O distance related to peroxo‐like dimers using the neutron pair distribution function. Recently, House et al.^[^ ^24^ ^]^ reported that the low voltage hysteresis of anionic redox reaction could be achieved by controlling superstructure between Li and Mn ions. By comparing two P2‐type ribbon‐ordered Na_0.60_Li_0.20_Mn_0.80_O_2_ and honeycomb‐ordered Na_0.75_Li_0.25_Mn_0.75_O_2_, they suggested that the ribbon‐type Li/Mn ordering inhibits disorder of the inherent patterning along the ab plane that causes voltage hysteresis by changing Mn coordination of O^n−^ ions.

Although the reversible anion‐based redox reactions with the small voltage polarization at the high‐voltage range during the first charge/discharge process have successfully occurred, severe decreases of the initial large anionic capacity originating from O^2−^/O^n−^ are observed upon cycling; and, how to innovate the unstable cycle retention arising from the anion‐redox‐induced two‐phase reaction lacks systematic studies so far. In addition, an in‐depth understanding of the two‐phase reaction is highly required to use the full potential of anion‐redox features because the unmixing reaction accompanied by huge structural variations for SIBs is a dominantly deteriorative point to the electrochemical cycle performance. Therefore, in this work, various experimental analyses combined with first‐principle calculations were performed to provide a comprehensive understanding of the anion‐redox‐induced two‐phase reaction at the high‐voltage (≈4.2 V vs Na^+^/Na) in the P2‐type Na_0.60_Li_0.20_Mn_0.80_O_2_ (NLMO). In addition, this study suggests rational design principles for enabling a practical use with a stable cycle feature of the Mn‐based layered oxide for SIBs. Our electrochemical charge–discharge profiles of NLMO unambiguously show the highly reversibility of two‐phase reaction induced by the anionic redox reaction of lattice O^2−^/O^n−^ at the high‐voltage. Using in situ synchrotron X‐ray diffraction (XRD) measurement, we clarify the unmixing phase reactions exhibiting the newly discovered Z phase evolution resulting from the original P2‐phase of NLMO during the charge/discharge process, and discovery the involvement of anionic redox reactions to compensate the charge‐imbalance caused by the Na‐extraction in the compound through ex situ soft X‐ray absorption spectroscopy (XAS) and X‐ray photoelectron spectroscopy (XPS). From the first‐principle calculation results, the detailed qualitative and quantitative calculations of Mn 3d‐electron and O 2p‐electron theoretically explain the oxygen redox mechanism without the further oxidation of Mn^4+^ to Mn^5+^ in the high‐redox‐potential of NLMO. Furthermore, the thermodynamic mixing entalpy energy clearly shows the distinct two‐phase reaction with the calculated desodiation potential during the charge process. Considering our concrete understandings on the anion‐redox‐induced unmixing reaction of Na‐rich and Na‐poor phases, reducing the degree of phase separation through the increase of vacancy‐solubility during the two‐phase reaction, and alleviating the volume shrinkage accompanied by the lattice mismatch between the separated phases are suggested to rationally use the highly reversible anion‐based redox reaction featuring high‐energy‐density coupled with stable cycle retention performance in Mn‐based layerd cathode materials for SIBs.

## 2 Results and Discussion

### 2.1 Electrochemical Characterizations of NLMO

**Figure 1:**
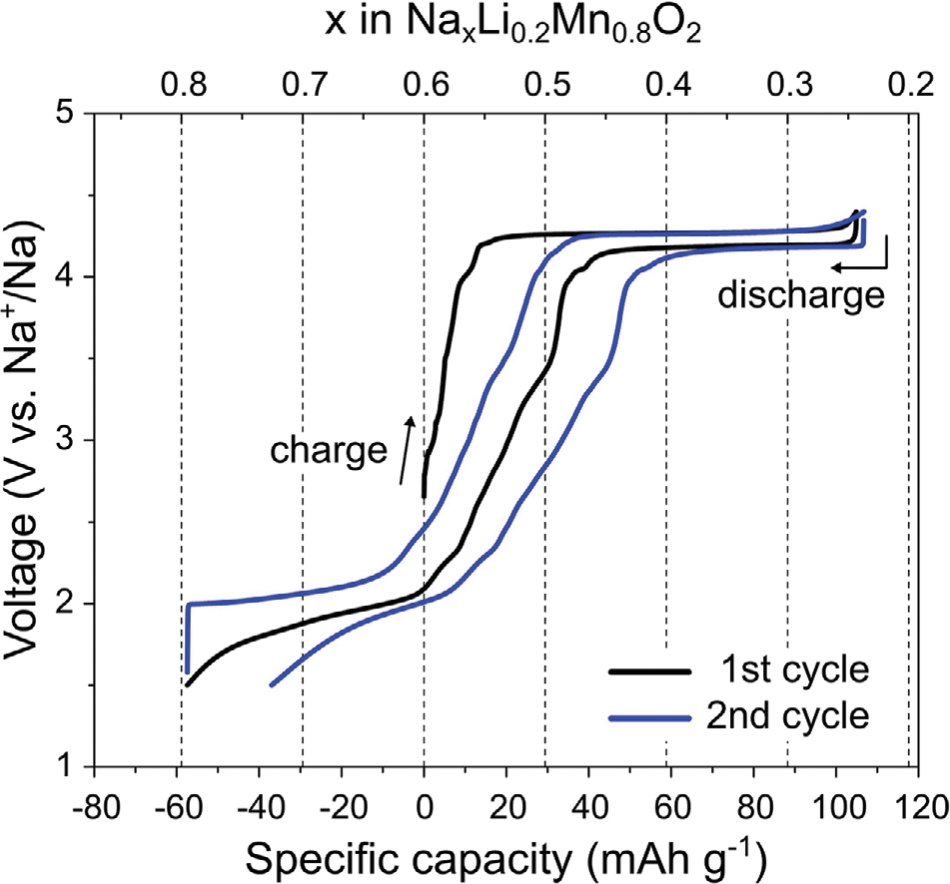
Galvanostatic charge/discharge profiles of NLMO during the first and second cycles. A current density of 10 mA g^−1^ was applied in the voltage range of 1.5–4.4 V.

P2‐type NLMO powder was synthesized via a solid‐state reaction. The major Bragg peaks of NLMO are clearly indexed as a P2‐type layered Na*_x_*MnO_2_ (S. G. P6_3_/mmc) as shown in Figure S1a, Supporting Information. The lattice parameters of NLMO are refined as *a* = 2.8492(1) and *c* = 11.1372(6). The corresponding P2‐type model is illustrated in Figure S1b, Supporting Information, where P indicates that Na ions are located at the prismatic sites between the edge‐sharing MnO_2_ layers, and the following number, 2, means that two MnO_2_ layers (AB and BA layers) are repeated along the c‐axis. In addition, the minor Bragg peaks at 12.8°, 20.0°, 21.3°, 26.6°, and 29.1°, which are not originated from the P2‐type structure, are consistent with peaks of the ribbon‐type Li/Mn ordering model (S. G. P2_1_/c) proposed in the previous report.^[^ ^24^ ^]^ The atomic composition of NLMO was evaluated as 0.594: 0.194: 0.806 (Na: Li: Mn) by using ICP‐AES. Figure S2, Supporting Information, presents the field emission scanning electron microscopy (FE‐SEM) image of synthesized powder, in which the sample has a homogeneous atomic distribution of Na, O, and Mn without the aggregation of the elements. The galvanostatic charge/discharge profiles of NLMO are shown in **Figure** **1**. NLMO exhibited a flat plateau at ≈4.26 V during the first charge process, indicating that it undergoes a two‐phase reaction during Na^+^ extraction. The initial charge capacity of NLMO up to 4.4 V is 105 mAh g^−1^, which corresponds to the extraction of approximately 0.36 Na^+^ per formula unit from its lattice. During the following discharge process, the high‐voltage plateau was reversibly maintained until ≈0.25 Na^+^ ions per formula unit (74 mAh g^−1^) were inserted into its lattice between 3.5 and 4.4 V. Since further oxidation of Mn^4+^ are not feasible at octahedral symmetry, the high voltage plateau is expected from reversible oxygen redox reaction. The corresponding charge compensation mechanism will be discussed later section. When discharged to 2 V, the Na^+^ content of NLMO returned to 0.6, which is identical to the initial sodium content. Moreover, NLMO exhibits a mild sloped profile between 2 and 1.5 V for additional Na^+^ insertion (0.2 Na^+^ per formula unit), which is expected to be associated with Mn^4+^ reduction.

### 2.2 Structural Evolution of NLMO upon Charge/Discharge Process

**Figure 2:**
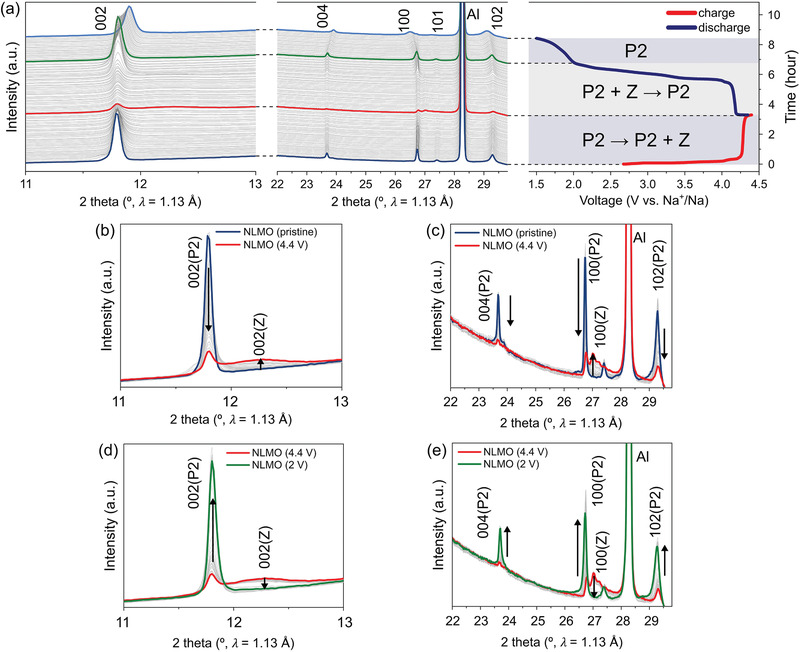
In situ synchrotron XRD analysis of Na/NLMO cell. a) Structural evolution of NLMO via in situ synchrotron XRD analysis during the first charge/discharge process at a rate of 30 mA g^−1^ in the voltage range of 1.5–4.4 V with the corresponding voltage profile. In situ XRD patterns collected during the first b,c) charge and d,e) discharge process of NLMO cell in the vicinity of the (002) peak and (004), (100), (101), and (102) peaks. The high‐intensity peak at 28.3° is attributed to an Al foil.

To investigate the detailed structural evolution of NLMO upon a galvanostatic charge/discharge test, in situ synchrotron XRD measurement was carried out during the first cycle, as shown in **Figure** **2**a. NLMO exhibited a typical two‐phase reaction behavior during the first charge process, as shown in Figure 2b,c. Accordingly, it was confirmed that all the intensities of the assigned peaks of NLMO, including the (002) and (004) diffraction peaks, gradually decreased without any change in peak positions, and a new set of diffraction peaks, including those at 12.2° and 27.0°, grew gradually. In comparison with the original phase, a newly evolved phase induced by Na^+^ extraction is characterized by a low‐crystalline structure derived from a considerable amount of stacking faults, reflected as very broad peaks. In particular, the peak broadening caused by stacking faults is more pronounced in the (002) diffraction peak than in the (100) diffraction peak, indicating relatively well‐preserved TMO_2_ planes and a severe loss of P2‐type stacking. This structural feature of the new phase is crystallographically similar to the highly desodiated P2‐type layered oxide materials referred to as the “Z” phase.^[^ ^25^, ^26^, ^27^ ^]^ Therefore, we will refer to this new phase as the Z phase for simplicity.

In Figure 2b, the new broad peak of the Z phase at 12.2° that were evolved at a higher angle than the initial P2 (002) peak at 11.8° confirmed that the Z phase after the sodium ion extraction has a contracted structure along the c‐axis compared to the initial P2 structure. This contraction during the P2‐Z phase transition may result from the migration of a metal ion from the TM layer to the empty site in the Na layer when the number of vacant Na ion sites is large.^[^ ^25^ ^]^ This migration is further confirmed by the evolution of X‐ray absorption near edge structure (XANES) Mn pre‐edge peaks during charge (Figure S4, Supporting Information). In addition, the new (100) diffraction peaks of the Z phase were split into two peaks at 27.0° and 27.2° with smaller d‐spacing values than that of NLMO, indicating that the Z phase also contracted along the ab plane (Figure 2c). When discharged to 2 V, NLMO returned to its initial structure via a two‐phase reaction pathway, as shown in Figure 2d,e. For additional Na^+^ insertion (discharging from 2 to 1.5 V), NLMO shows solid‐solution behavior as can be seen in Figure 2a.

### 2.3 Spectroscopic Study and Charge Compensation Mechanism of NLMO

**Figure 3:**
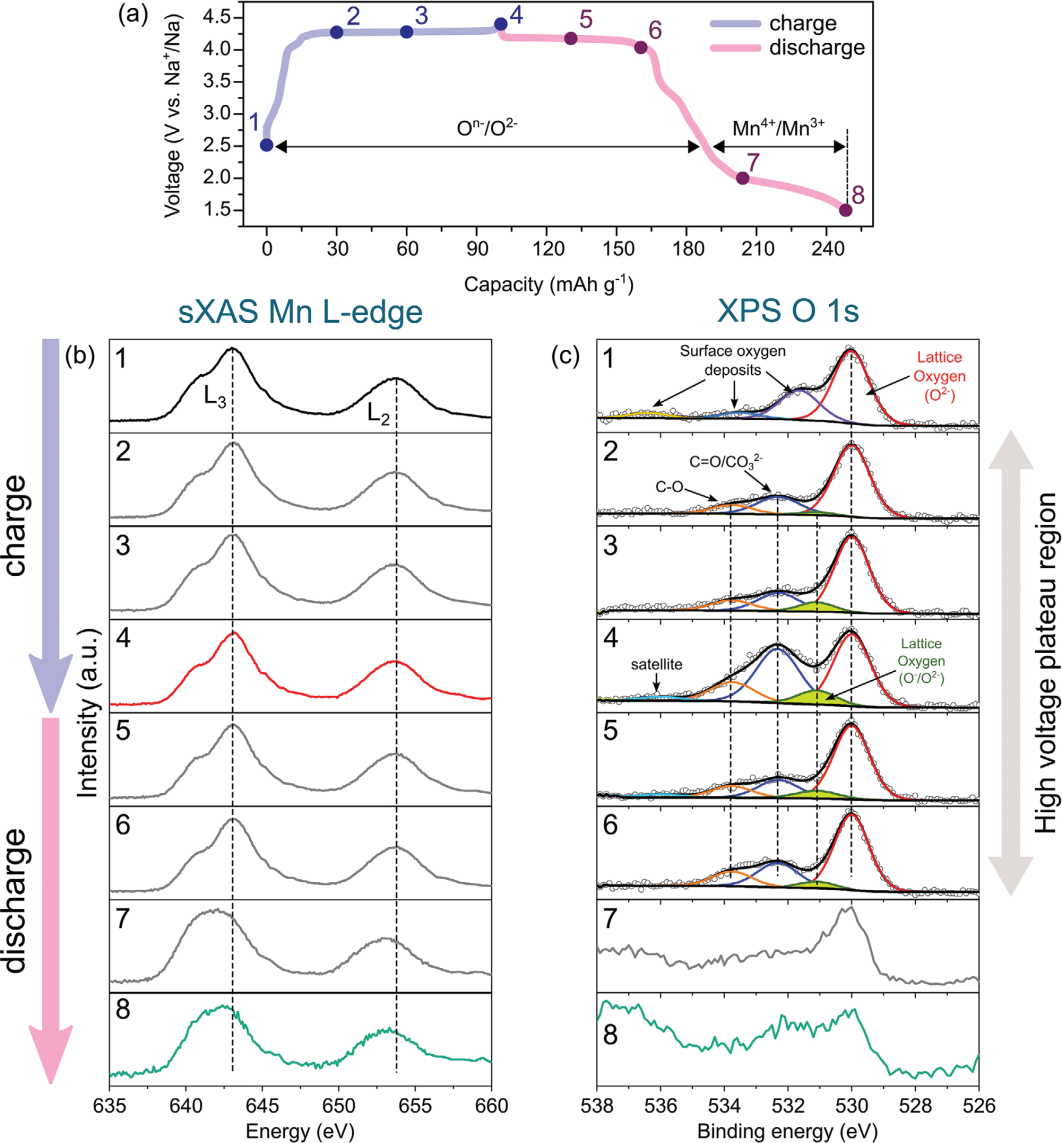
Ex situ sXAS and XPS results of NLMO. a) Charge/discharge profiles of NLMO during the first cycle at a rate of 30 mA g^−1^. Evolution of b) sXAS Mn L‐edge spectra measured in PEY mode and c) XPS O 1s spectra corresponding to the points in Figure 5a.

To confirm the charge compensation mechanism of NLMO, ex situ soft XAS (sXAS) at the Mn L‐edge spectra and Ar etching assisted ex situ XPS at the O 1s spectra were employed for cationic and anionic redox couple, respectively (**Figure** **3**). Although sXAS using a partial electron yield (PEY) mode provides surface‐sensitive information (≈1 nm), the changes in the Mn oxidation state can be clearly investigated in the ex situ sXAS at Mn L‐edge spectra because the X‐ray absorption peaks corresponding to the metal L_2,3_‐edge are relatively intense and very sensitive to the oxidation state of the metal owing to the electric dipole‐allowed 2p → 3d transition.^[^ ^28^ ^]^ Figure 3b shows the evolution of the Mn L_2,3_‐edge spectra measured at various charged and discharged states corresponding to the points in Figure 3a. The two main peaks for Mn, the L_3_‐edge at 643.1 eV and L_2_‐edge at 653.8 eV, are caused by the respective electronic transitions from the 2p_3/2_ and 2p_1/2_ core levels to an unoccupied 3d level. Moreover, the split L_3_‐edge feature of pristine state is highly consistent with that of MnO_2_ in a previous report.^[^ ^29^ ^]^ The peak position of the Mn L_2,3_‐edge did not change throughout the first charge process, indicating that the tetravalent Mn^4+^ of the pristine state did not contribute to the charge compensation due to the difficulty in further oxidation of Mn^4+^ at the octahedral site. In addition, during the subsequent discharge process, this oxidation state was almost constant until the end of the oxygen redox plateau (No. 6 in Figure 3a). Therefore, charge compensation of NLMO during the charge process is expected to be induced by O^n−^/O^2−^ (*n* < 2) on the lattice oxygen of the crystalline network.

However, NLMO exhibited a significant shift in its peak position to a lower energy level when discharged at 2 V, and this was accompanied by a change in the peak shape, which has no split feature similar to Mn_2_O_3_.^[^ ^29^ ^]^ Thus, in this region, the reduction reaction from Mn^4+^ to Mn^3+^ contributed to the charge compensation. These results are well consistent with in situ Mn K‐edge XANES results, in which the Mn K‐edge spectra gradually shifted to a lower energy level after the high voltage plateau (from No. 9 to No. 12), as shown in Figure S3.

Additionally, in the K‐edge XANES spectrum of transition metals, the pre‐edge peaks are strongly related to the coordination number; the four‐coordinate state has a larger pre‐peak intensity than the six‐coordinate state.^[^ ^30^ ^]^ Figure S4 illustrates the enlarged pre‐edge region corresponding to the Mn K‐edge XANES spectra of pristine, charged (4.4 V), and discharged (1.5 V) states of NLMO. The pre‐edge peaks in the Mn K‐edge spectra consist of two distinct peaks at 6,541 eV due to the electric transition from the 1s orbital to the *t*_2g_ orbitals, and 6,543 eV due to the electric transition from the 1s orbitals to the *e*_g_ orbitals. During the charge process of NLMO, the intensity of the pre‐edge peaks increased, as shown in Figure S4. This increased intensity at high voltage supports the migration of a fraction of Mn ions in the TM layer into the tetrahedral sites of the Na layer. During the subsequent discharge process, the pre‐peak intensity again decreased at 1.5 V. However, the pre‐peak intensity did not completely return to its original state. These results indicate that the metal migration is only partially reversible during the two‐phase reaction and the residual Mn ions remains at the tetrahedral sites.

To examine the contribution of oxygen redox to the charge compensation of NLMO, we carried out ex situ XPS at the O 1s region, as shown in Figure 3c. The O 1s spectra of the pristine state consist of four components. A strong peak at the lowest binding energy (BE) of 530.0 eV is assigned as O^2−^ anions from the crystalline network, whereas other peaks at higher BEs are ascribed to contamination by surface oxygen deposits. Upon charging, new three peaks were evolved besides the lattice oxygen (O^2−^) peak. The two major peaks at 532.3 and 533.8 eV are attributed to CEI components, which correspond to carbonates/O—C=O and C—O in O—C=O, respectively.^[^ ^31^ ^]^ Another minor peak at 531.1 eV was introduced to account for the asymmetry of the lattice oxygen peak, which has an increased BE of ≈1 eV compared to that of the lattice oxygen peak. This extra peak is attributed to the oxidized oxygen O^n−^ (*n* < 2) formed by the oxidation of lattice oxygen during charging at high voltage, according to the previous reports.^[^ ^18^, ^20^, ^32^, ^33^ ^]^ From these observations, it is confirmed that the lattice oxygen of NLMO solely contributes the charge compensation upon charging without any participation of the Mn ion oxidation. The relative intensity of the O^n−^ peak gradually increased up to the end of charging as compared to the lattice oxygen (O^2−^) peak. Subsequently, the intensity of O^n−^ peak gradually decreased over the high‐voltage plateau region where discharging proceeded, indicating that oxygen reversibly reduced during discharge. Therefore, the charge compensation of NLMO is contributed by the O^n−^/O^2−^ redox reaction in the high‐voltage plateau region and Mn^4+^/Mn^3+^ redox reaction in the sloped region, respectively.

### 2.4 Anion‐Redox‐Reaction Induced Two‐Phase Reaction

**Figure 4:**
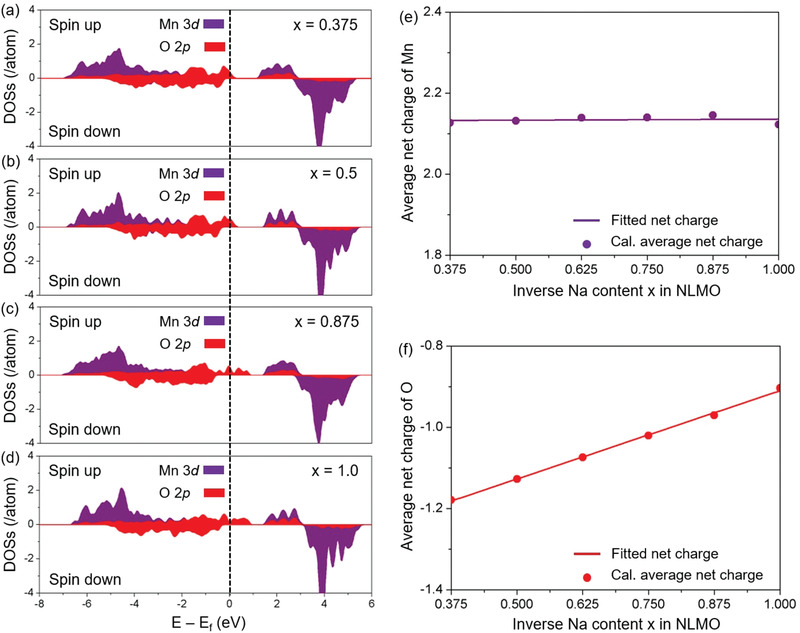
Qualitative and quantitative electron‐structure analyses of Mn and O. Combined partial density of states (PDOSs) of Mn 3d‐electron (purple) and O 2p‐electron (red) at a) *x* = 0.375, b) 0.5, c) 0.875, and d) 1.0 in NLMO. Average net charges obtained from the Bader charge calculation for e) Mn and f) O and their fitted values as a function of inverse Na content (*x*) in NLMO.

To theoretically understand the in‐depth redox mechanism in NLMO during desodiation, detailed qualitative and quantitative electronic structures were examined using first‐principle calculations. **Figure** **4**a‐d indicate combined graphs of the partial density of states (PDOSs) of Mn 3d‐electron and O 2p‐electron as a function of inverse Na content (*x*) in NLMO over the charge range (0.375 ≤ *x* ≤ 1.0). Based on the crystal field theory (CFT), Figure 4a shows that PDOSs of NLMO at *x* = 0.375 presents a conventional charge order of Mn with the oxidation state of 4+ at high‐spin states (*t*_2g_^3^ *e*_g_^0^), similar with the pristine NLMO sample. In general, a further oxidation of Mn^4+^ to Mn^5+^ is not preferential at the octahedral site coordinated with six oxygen in the perspective of energetics in CFT; therefore, the stabilized electronic configuration of Mn^4+^ predicts that the redox activity from NLMO would be generated by the anionic redox reaction of O^2−^/O^n−^, because some O 2p‐electron are located in higher energy states as compared with the stabilized Mn 3d‐electron in lower energy states. Figure 4b–d illustrate the variations of Mn 3d‐electron and O 2p‐electron during the charge. All Mn PDOSs are deemed as the stabilized Mn^4+^ electronic configuration, whereas an oxidation behavior of O 2p‐electron is observed over the charge range. These qualitative analyses imply that the anion species participate in the compensation of charge‐imbalance when Na is extracted. For a quantitatively direct investigation of the redox mechanism, we calculated the net charges, calculated by the Bader charge method, of Mn and O during the Na‐extraction (see Figure 4e,f). Furthermore, the calculated average net charges are carefully fitted using a first order polynomial for a clear understanding. All Mn values are similar each other, while a linear increase trend of O net charge value is examined during the charge process. This distinct difference between Mn and O is consistent with the above observed PDOSs of cation and anion electrons, which identifies the anion‐based redox reaction of O^2−^/O^n−^.

**Figure 5:**
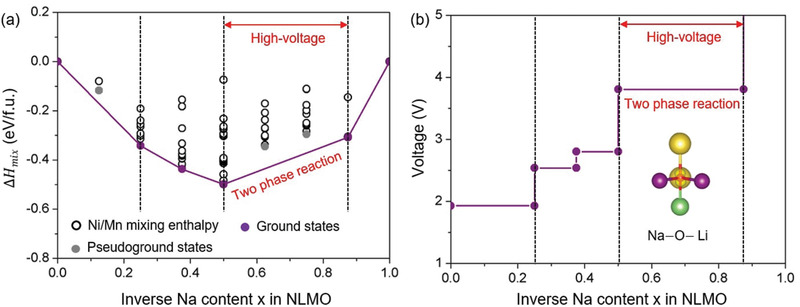
Thermodynamic phase‐stability and desodiation potential. a) Formation energies of mixing enthalpy considering all possible □(vacancy)/Na atomic configuration based on a binary mixture of the fully sodiated (NLMO) and desodiated phases (LMO) as a function of □ content (*x*) in NLMO. b) Computationally calculated voltages with varying *x* in NLMO. The inset indicates local spatial electron distribution in the valence band from −2 to 0 eV (Fermi level) in the desodiated NLMO.

On the basis of the anion‐redox‐reaction induced two‐phase reaction, observed by the various experimental measurements, we calculated the formation energy of mixing enthalpy in light of all possible □(vacancy)/Na atomic configurations as a function of □ content (*x*) in NLMO over the full range for 0.0 ≤ *x* ≤ 1.0 so that it is to investigate in detail a thermodynamic phase‐stability during the Na‐extraction (**Figure** **5**a). From the thermodynamic viewpoint, this energy diagram suggests that □ is soluble into all phases over the full range for 0 ≤ *x* ≤ 1 in NLMO (this can be elucidated by Δ*H*_mix_ < 0) For a better understanding, the lowest‐energy phases at each ratio of □/Na were determined to be ground (*x* = 0.0, 0.25, 0.375, 0.5, 0.875, and 1.0) and pseudoground (*x* = 0.125, 0.625, and 0.75) states, as depicted in Figure 5a. Based on a phase diagram of a binary mixture consisting of fully sodiated and desodiated phases, the mixing enthalpy value reveals that the ground states lying on the tie line between *x* = 0.5 and *x* = 0.875 in NLMO indicating a distinct two‐phase reaction, which is consistent with the experimentally observed two‐phase XRD pattern and electrochemical charge profile. In the viewpoint of thermodynamics, a □‐solubility into NLMO decreases for 0.5 ≤ *x* ≤ 0.875; that is, a relative phase‐stability NLMO decreases with varying □ content (*x*) during the charge process. These thermodynamic phase‐instability originate from the anion‐based redox reaction of O^2−^/O^n−^ when Na is extracted from the oxides in the charge range. In addition, a further Na‐extraction from *x* = 0.875 to *x* = 1.0 leads to the thermodynamically drastic decrease of phase‐stability resulting in significant irreversibility of capacity, and voltage‐drop in the first cycle, which is not favorable region to gain electrochemical high‐performance upon cycling. Using the thermodynamic mixing enthalpy values with varying □ content (*x*), we calculated desodiation potentials of NLMO during the charge process (Figure 5b), and their results are well consistent with the experimentally measured charge‐profile. In addition, we identified the isolated O 2p‐electron of Na—O—Li linear configuration in the anion‐redox‐induced two‐phase reaction based on a spatial electron distribution calculation (see the inset of Figure 5b), which have been considered as the origin of oxygen redox reaction in various Na‐based layered compounds.

Although our experimental and theoretical findings have showed the highly reversible two‐phase reaction induced by the anionic redox reaction in the high‐voltage (≈ 4.2 V vs Na^+^/Na) for NLMO as a promising cathode material for SIBs, the reversible capacity of two‐phase reaction originating from the anion‐based reaction of O^2−^/O^n−^ gradually decreases upon charge/discharge cycling. Thus, despite the stable capacity retention of NLMO, it suffers from a gradual voltage decay accompanied by a reduction of high voltage plateau resulting in a sloped discharge curve similar to various anionic redox active layered material, which would be correlated with the gradual structural rearrangement due to the accumulation of Mn ions at the tetrahedral of Na layer during the subsequent cycles. Therefore, clarified insights are required to enable high‐energy‐density with a cycle stability of NLMO upon cycling. First, from the perspective of thermodynamics, the two‐phase reaction in the high‐voltage region generates a severe phase separation into an electrochemically active Na‐rich and inactive Na‐poor phases owing to the thermodynamic instability in a spinodal decomposition region. Second, from the chemomechanics viewpoint, a drastic volumetric shrinkage accompanied by the thermodynamic phase separation would result in inter‐ and intra‐granular cracks derived from mismatch strains between separated phases and an acceleration of the evolution of the phase separation reaction. Finally, for a rational use of the highly reversible two‐phase reaction, occurring the high‐voltage at ≈4.2 V (vs Na^+^/Na), induced by the anion‐redox‐reaction upon cycling, we suggest that A‐pillared NLMO cathode materials (A: mono and divalent species featuring a rigid element during charge/discharge) exhibiting i) a reduction of the degree of phase separation based on the increase of □‐solubility into the pillared layered oxide and ii) a physical suppression of the extreme structural deformation upon cycling.

## 3 Conclusion

The highly reversible anion‐redox‐induced two‐phase reaction operating at the high voltage (≈4.2 V vs Na^+^/Na) in NLMO cathode material was explored using various experimental analyses such as in situ synchrotron XRD, ex situ soft XAS, and XPS and first‐principle calculations considering cation‐anion‐combined electronic structures and thermodynamics. The electrochemical charge‐discharge profile of P2‐type NLMO showed the preserved high‐potential plateau during the charge and subsequent discharge processes, which was understood by the measurements of in situ synchrotron XRD patterns presenting the newly evolved Z‐phase (Na‐poor) coexisting with the original P2‐phase (Na‐rich) in the structural perspective. From the various spectroscopic investigations, the anionic redox reaction induced by the lattice O^2−^/O^n−^ during the thermodynamic unmixing reaction in the oxide was confirmed without any further oxidation of Mn^4+^ to Mn^5+^. These experimental results were in detail elucidated by the combined electronic structures of Mn 3d‐electron and O 2p‐electron based on the energetics of CFT, the thermodynamic phase‐stability considering the binary mixture of fully sodated and desodiated phases, and the theoretically calculated desodiation potentials. In spite of the surprising reversibility of oxygen redox activity exhibiting the high (de)sodiation potential for high‐energy‐density cathode materials, the two‐phase reaction induced by the oxygen‐redox showed the drastic decrease of reversible anion‐capacity upon cycling. Therefore, we believe that a breakthrough approach to maintain the reversible anion‐capacity with substantial cyclability may be easily found from the thermodyanamic and chemomechanical perspectives such as i) lowering a spinodal decomposition barrier closely related to the vacancy‐solubility and ii) reducing the severe volumetric strain coupled with the residual strain caused by the separated phases for Mn‐based layered cathode materials in SIBs. Furthermore, this work illustrates an intriguing material design concept for the direct utilization of the high‐voltage redox plateau in anionic redox‐active layered compounds for rechargeable battery research fields.

## 4 Experimental Section

##### Material Synthesis

P2‐type Na_0.6_Li_0.2_Mn_0.8_O_2_ powder was synthesized through a solid‐state reaction with Na_2_CO_3_ (Kanto chemical), Li_2_CO_3_ (Alfa Aesar), and Mn_2_O_3_ (Sigma Aldrich) as the metallic precursors. Accurate stoichiometric amounts of the precursor powders, without excess Na or Li sources, were homogeneously mixed via ball milling with acetone. After drying at 65 °C for 2 h, the powder mixture was heat‐treated in air at 900 °C for 10 h and was naturally allowed to cool to room temperature. To avoid unfavorable side reactions with the ambient atmosphere and moisture, the synthesized powder was transferred to an argon‐filled glove box after the heat treatment.

##### Structure Analysis

XRD patterns of the sample powders were collected using a SmartLab (Rigaku, Japan) with Cu K*α* radiation (*λ* = 1.5406 Å) in the 2ϑ range from 10° to 100°. The Rietveld refinement of XRD pattern was carried out using general structure analysis system software.^[^ ^34^ ^]^ The schematic illustration of P2‐type layered structure was obtained using visualization for electronic and structural analysis software.^[^ ^35^ ^]^ Inductively coupled plasma atomic emission spectroscopy (ICP‐AES) was performed to obtain the atomic composition of each sample using OPTIMA 8300 (Perkin‐Elmer, USA). FE‐SEM images were collected using AURIGA (Zeiss, Germany).

##### Electrochemical Method

Slurry containing the synthesized P2‐type Na_0.6_Li_0.2_Mn_0.8_O_2_ was prepared by homogeneously mixing the active material powder, Super P, and polyvinylidene fluoride in *N*‐methylpyrrolidone in a weight ratio of 7:2:1. This slurry was cast on an Al foil and dried at 120 °C for 10 h in a vacuum. The thoroughly dried composite electrode was roll‐pressed and transferred to the argon‐filled glove box. For the electrochemical evaluations, 2032‐type coin cells were assembled, in which a single sodium metal disk (Sigma Aldrich) was used as the counter and reference electrodes, and 1 m NaClO_4_ in ethylene carbonate (EC)/propylene carbonate (PC) (1:1 v/v) with 2 wt% fluoroethylene carbonate as an additive was used as the electrolyte. Glass fiber (Whatman GF‐A) was used as a separator. Galvanostatic charge/discharge cycles at various current densities were performed at 25 °C on a WBCS3000S battery cycler (WonAtech, Korea). The cells were cycled at a current density of 10 mA g^−1^ between 1.5 and 4.4 V (vs Na^+^/Na). All the voltage units (V) in this work indicate the measured voltages using sodium metal as the counter electrode (V vs Na^+^/Na).

##### In Situ Synchrotron XRD Analysis

In situ synchrotron XRD measurement was performed on the beamline 3D at Pohang Light Source II (PLS II), Korea. To use the transmission measurement mode, a customized 2032‐type coin cell with a hole in the center was employed. Data were collected on a Mar 345 image plate (marXperts GmbH, Germany) with incident wavelength of 1.1273 Å for P2‐type Na_0.60_Li_0.20_Mn_0.80_O_2_. XRD patterns were obtained at intervals of approximately 5 min. The 2D diffraction images were radially integrated using Fit2D software.^[^ ^36^ ^]^

##### In Situ and Ex Situ X‐Ray Absorption Spectroscopy

In situ X‐ray absorption spectroscopy (in situ XAS) measurement was performed on the beamline 8C at Pohang Light Source II (PLS II), Korea. The incident beam was tapered by an in‐vacuum undulator, and a Si(111) double‐crystal monochromator was used to eliminate higher‐order harmonics. Similar to the in situ synchrotron XRD measurements, the customized 2032‐type coin cell was used for applying a galvanostatic charge/discharge test. Mn K‐edge spectra were collected under the transmission mode using gas ionization chambers to measure the incident and transmitted X‐ray intensities, and a Mn foil was used as an internal reference for the photon energy calibration. Data analysis was carried out using an Athena software package.^[^ ^37^ ^]^ Li_2_MnO_3_, Mn_2_O_3_, and MnO_2_ powders were used as references. Ex situ soft X‐ray absorption spectroscopy (ex situ sXAS) measurements (Mn L_2,3_‐edge) were carried out on beamline 4D at PLS II. After careful disassembly of the P2‐type Na_0.6_Li_0.2_Mn_0.8_O_2_ cells with different charged/discharged states in an argon‐filled glove box, the electrodes were rinsed with a pure propylene carbonate solvent several times over to remove any residual Na salts and dried in a vacuum chamber of the glove box. To avoid exposure to air, an argon‐filled laboratory‐made vessel was used for transferring the samples into the ultra‐high‐vacuum (<10^−9^ Torr) chamber of the synchrotron X‐ray equipment. The absorption spectra were collected with the PEY mode, which has a probing depth of ≈1 nm.

##### Ex Situ X‐Ray Photoelectron Spectroscopy

XPS measurements were carried out using Sigma Probe (ThermoFisher Scientific, UK) with Al K*α* (1486.8 eV) as the X‐ray source. Samples of the P2‐type Na_0.6_Li_0.2_Mn_0.8_O_2_ cells for XPS measurements were prepared in the same manner with ex situ sXAS. To avoid air exposure, coin cells were disassembled in Ar‐filled glove box, which was directly connected to XPS sample chamber. Each sample was analyzed after etching using Ar ion beam for about 60 s. Peaks were recorded with a constant pass energy of 20 eV. The binding energy scale was calibrated using the C 1s core peak at 284.6 eV from the Super P, which was used in the preparation of the electrodes. XPS O 1s spectra were analyzed using a Shirley‐type background and 70% Gaussian/30% Lorentzian line shapes. The peak positions and areas were optimized by a weighted least‐squares method.

##### First‐Principle Calculation

Based on a plane wave set with projector augmented wave pseudopotentials, a density functional theory method implemented in the Vienna ab initio simulation package was employed to conduct all theoretical calculations. The generalized gradient approximation utilizing the Perdew–Burke–Ernzerhof model was used for the exchange‐correlation functional with spin‐polarized conditions. In light of the strong interaction between the 3d‐band of transition metals and the 2p‐band of ligands in crystal frameworks, a Hubbard‐type U correction applied to the pseudopotentials in all calculations was used, and the corresponding value of Mn was taken from previous reports.^[^ ^38^ ^]^ To more accurately describe the Na^+^, the Na pseudopotential including one 3s‐ and six 2p‐electrons and the standard Mn potential were used. In the perspective of typical computational parameters, the Monkhorst‐Pack approach was covered to sample the *k*‐points in the reciprocal space with 4 × 4 × 2 meshes; in addition, a cut‐off energy of 400 eV was dealt in all calculations. To obtain the thermodynamic energies and the electronic structures in the ground states, the cell parameters and atomic coordinates were fully relaxed. The thermodynamic formation energy of mixing enthalpy considering binary mixtures of the fully (de)sodiated phases and the equilibrium average voltage during desodiation were calculated on the basis of the well‐developed model.^[^ ^39^, ^40^, ^41^ ^]^

## Conflict of Interest

The authors declare no conflict of interest.

## Supporting information

Supporting Information
