# Supplementary material for: Structural and Thermodynamic Understandings in Mn‐Based Sodium Layered Oxides during Anionic Redox
Source: Adv Sci (Weinh). 2020 Jul 2;7(16):2001263. doi: 10.1002/advs.202001263 (PMC7435253; doi:10.1002/advs.202001263)
Supplement: Supplementary file 1 — Supporting Information [file ADVS-7-2001263-s001.pdf]

Copyright WILEY-VCH Verlag GmbH & Co. KGaA, 69469 Weinheim, Germany, 2020.

Supporting Information

Structural and Thermodynamic Understandings in Mn-Based Sodium Layered Oxides during Anionic Redox

Seok Mun Kang†, Duho Kim†, Kug-Seung Lee, Min-Seob Kim, Aihua Jin, Jae-Hyuk Park, Chi-Yeong Ahn, Tae-Yeol Jeon, Young Hwa Jung, Seung-Ho Yu, Junyoung Mun,* and Yung-Eun Sung**

|

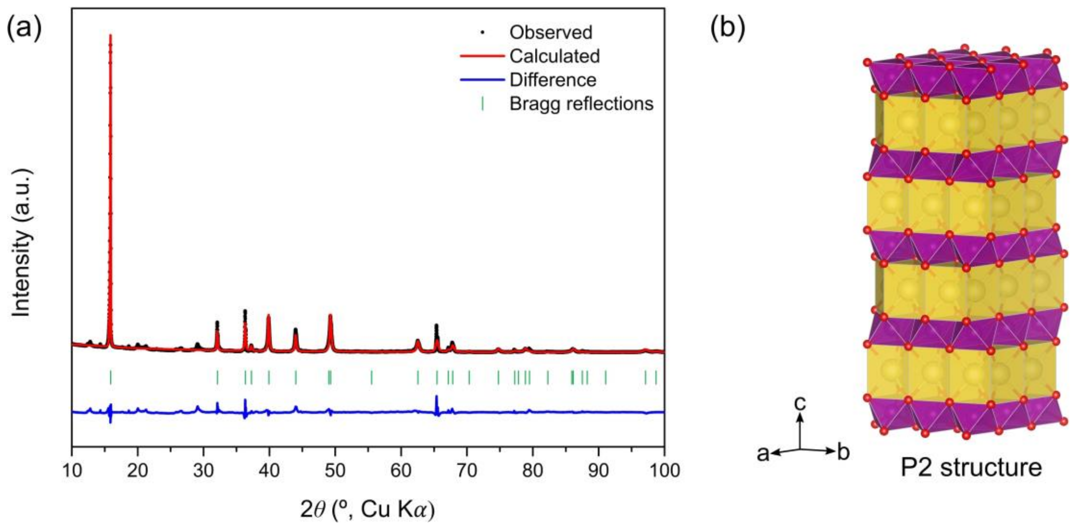

Figure S1. Structure analysis of NLMO. a) X-ray powder diffraction data for NLMO refined using b) the P2-type layered Na_xTMO_2 structure (S.G. $P6_3/mmc$). Rietveld refinement was carried out using GSAS software.

Table S1 Refined crystallographic parameters of NLMO from Rietveld refinement.

S. G. $P6_3/mmc$, *a* = b = 2.8492(1) Å, *c* = 11.1372(6) Å, V = 78.298(8) $Å^3$, R_{wp} = 2.90%

Site	Mult.	x	y	Z	Occ.	U_{iso}
**Mn1**	2	0	0	0	0.806[a]	0.0081(15)[b]
**Li1**	2	0	0	0	0.194[a]	0.0081(15)[b]
**O1**	4	1/3	2/3	0.088(2)	1	0.057(15)
**Na1**	2	1/3	2/3	1/4	0.28(4)[a]	0.041(14)[b]
**Na2**	2	0	0	1/4	0.32(4)[a]	0.041(14)[b]

a)The occupancies of Mn, Na, and Li are based on the ICP result.

b)U_{iso} values for each atom in the same layer are assumed to be equal.

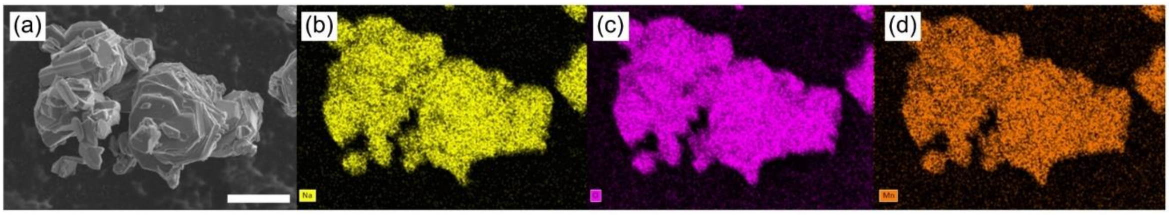

Figure S2. Morphology and elemental distributions of NLMO. a) FE-SEM image and elemental mapping of b) Na, c) O, and d) Mn for NLMO. The scale is 6 μm for all the individual images.

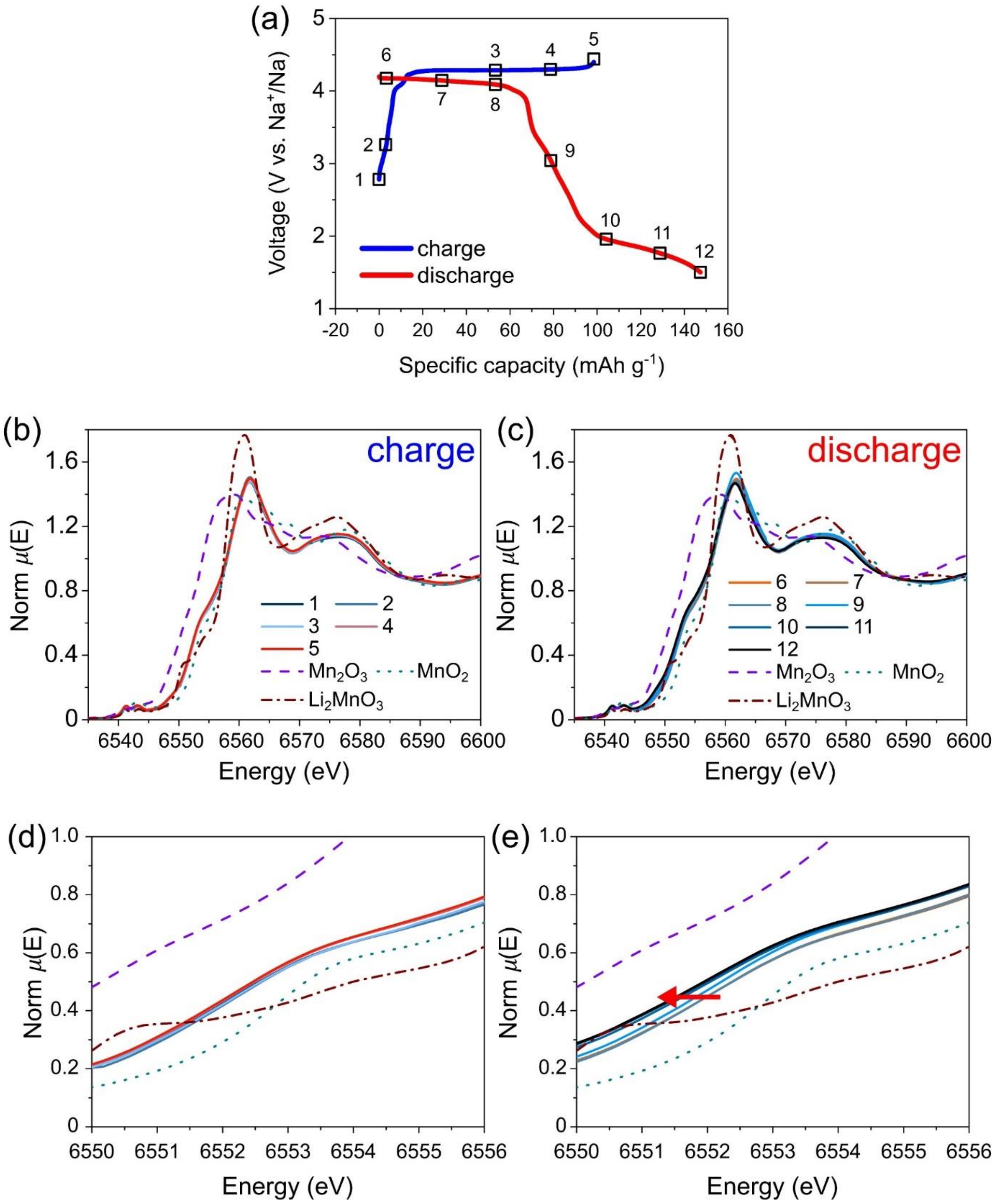

Figure S3. *In situ* XAS analysis at the Mn K-edge of NLMO during the first electrochemical cycle. a) The charge/discharge profile of Na/ NLMO cell at a rate of 30 mA g^{-1} between 1.5 and 4.4 V during the *in situ* measurement. Evolution of the Mn K-edge XANES spectra upon b) charging and c) discharging. d,e) Enlarged spectra at the edge region corresponding to the XANES spectra of b,c), respectively. Mn K-edge XANES spectra of Mn_2O_3 (Mn^{3+}), MnO_2 (Mn^{4+}), and Li_2MnO_3 (Mn^{4+}) are presented for reference. The numbers in Figure S3b, c, and f indicate the measurement points corresponding to those in Figure S3a.

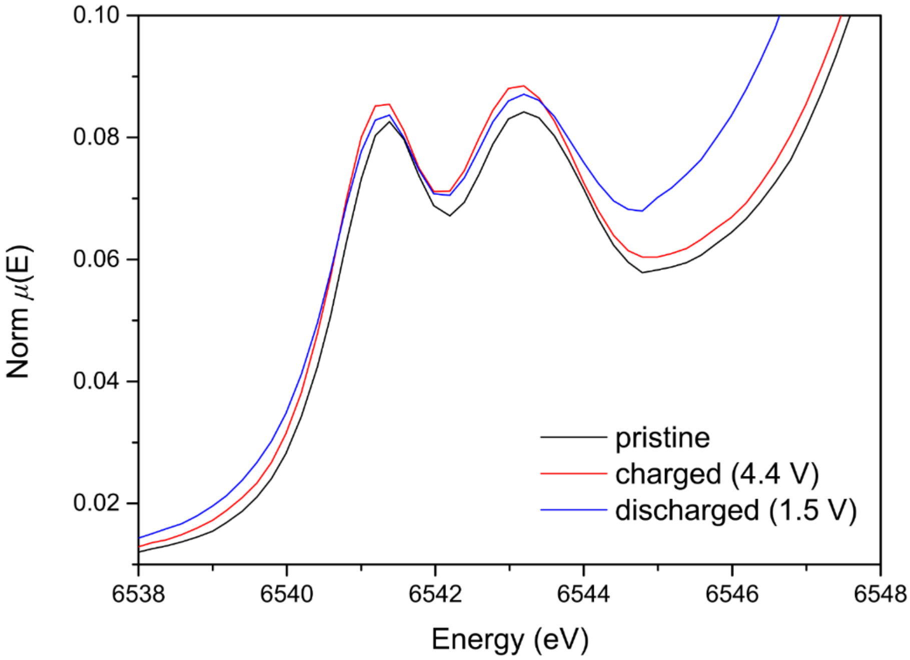

Figure S4. Comparison of the Mn K-edge pre-edge normalized XANES spectra of pristine, charged (4.4 V), and discharged (1.5 V) states of NLMO.

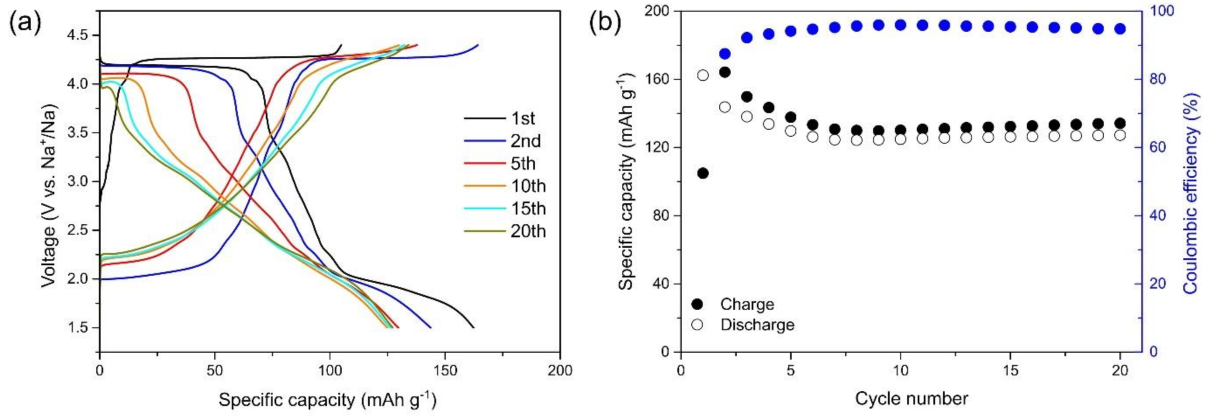

Figure S5. Electrochemical cycling performance of Na/NLMO cell. a) Charge/discharge profiles of NLMO during 20 cycles. b) Specific capacity and Coulombic efficiency of Na/NLMO cell as a function of cycle number.

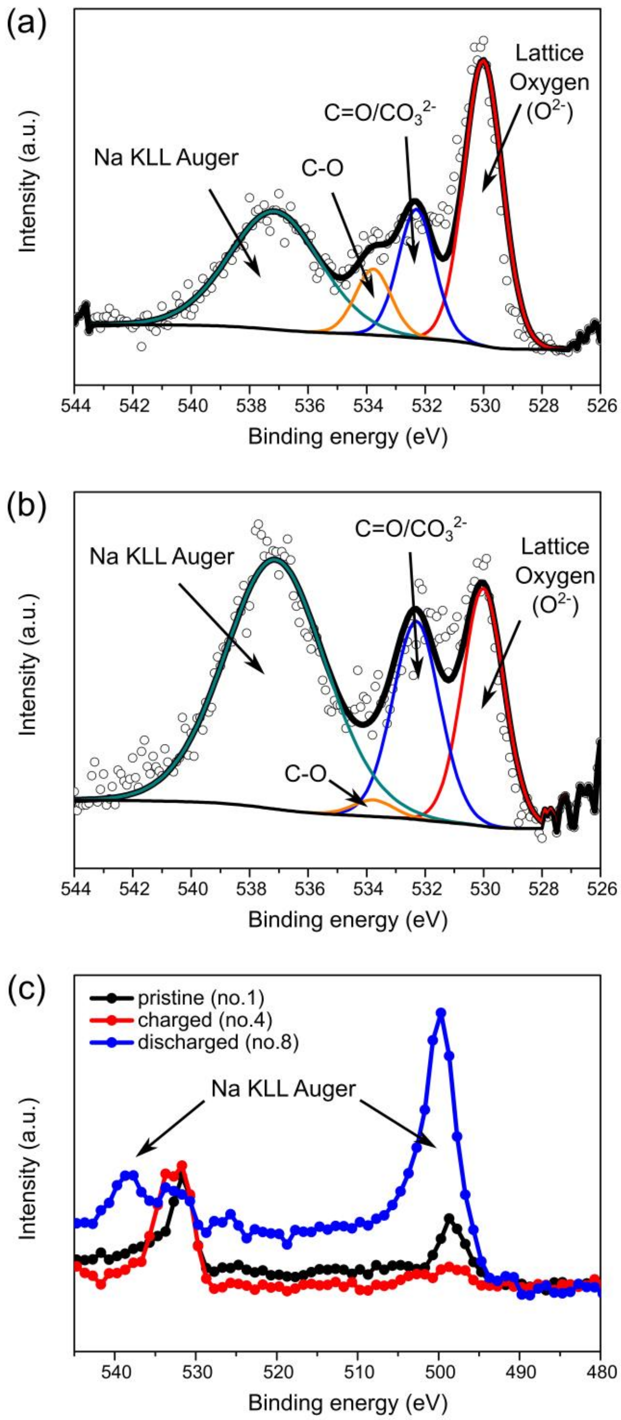

Figure S6. XPS O*1s* peak fitting results of a) no.7 and b) no.8 states in Figure 3. c) Evolution of Na KLL Auger peaks during the first charge and discharge process.
